# Integrated Profiling Identifies Long-Term Molecular Consequences of Prenatal Dexamethasone Treatment in the Rat Brain—Potential Triggers of Depressive Phenotype and Cognitive Impairment

**DOI:** 10.1007/s12035-024-04586-7

**Published:** 2024-11-11

**Authors:** Magdalena Kukla-Bartoszek, Marcin Piechota, Maciej Suski, Jacek Hajto, Małgorzata Borczyk, Agnieszka Basta-Kaim, Katarzyna Głombik

**Affiliations:** 1 Laboratory of Immunoendocrinology, Department of Experimental Neuroendocrinology, Polish Academy of Sciences, Maj Institute of Pharmacology, Smętna 12, 31-343 Kraków, Poland; 2 Laboratory of Pharmacogenomics, Department of Molecular Neuropharmacology, Maj Institute of Pharmacology, Polish Academy of Sciences, Smętna 12, 31-343 Kraków, Poland; 3 Department of Pharmacology, Jagiellonian University Medical College, Faculty of Medicine, Grzegórzecka 16, 31-531 Kraków, Poland; 4 Centre for the Development of Therapies for Civilization and Age-Related Diseases CDT-CARD, Jagiellonian University Medical College, Skawińska 8, 31-066 Kraków, Poland

**Keywords:** Depression, Glucocorticoids, High-throughput analysis, Brain

## Abstract

Prenatal excess of glucocorticoids (GCs) is considered to be one of the highly impacting factors contributing to depression development. Although GCs are crucial for normal fetal development and their administration (mainly dexamethasone, DEX) is a life-saving procedure for those at risk of preterm delivery, exposure to excess levels of GCs during pregnancy can yield detrimental consequences. Therefore, we aimed to systematically investigate the brain molecular alterations triggered by prenatal DEX administration. We used a rat model of depression based on prenatal exposure to DEX and performed integrative multi-level methylomic, transcriptomic, and proteomic analyses of adult rats’ brains (i.e., frontal cortex (FCx) and hippocampus (Hp)) to identify the outcomes of DEX action. Each of the investigated levels was significantly affected by DEX in the long-term manner. Particularly, we found 200 CpG islands to be differentially methylated in the FCx and 200 in the Hp of prenatally DEX-treated rats. Global transcriptomic analysis uncovered differential expression of transcripts mostly in FCx (271) and 1 in Hp, while proteomic study identified 146 differentially expressed proteins in FCx and 123 in Hp. Among the identified enriched molecular networks, we found altered pathways involved in synaptic plasticity (i.e., cAMP, calcium, and Wnt signaling pathways or tight junctions and adhesion molecules), which may contribute to cognitive impairment, observed in DEX-treated animals. Moreover, in the FCx, DEX administration in the prenatal period downregulates the expression of ribosome protein genes associated both with large and small ribosomal subunit assembly which can lead to a global decrease in translation and protein synthesis processes and, indirectly, alterations in the neurotransmission process.

**Supplementary Information:**

The online version contains supplementary material available at 10.1007/s12035-024-04586-7.

## Introduction

The prenatal and early postnatal periods of life are critical stages for proper organismal growth and development. It is a time marked by rapid cell proliferation and differentiation, making it particularly sensitive to early-life adversities that can yield detrimental effects [1]. Research indicates that adverse intrauterine conditions and maternal harmful behaviors, such as substance abuse, stress, and exposure to air pollutants and chemicals, as well as nutritional, psychological, and social conditions during early life, may not only trigger short-term negative effects but also program the offspring to significantly increased risk of chronic disease incidence in adult life [2–4].

Prenatal excess of glucocorticoids is one of the highly impacting risk factors associated with neuropsychiatric disorders development in adult life, and due to these detrimental effects, strict control of glucocorticoid levels is highly important. Glucocorticoids (GCs) are steroid hormones produced by the adrenal cortex in response to stress, but their concentration may also be physiologically increased, which happens during pregnancy [5]. A natural barrier constitutes placental 11β-hydroxysteroid dehydrogenase type 2 (11β-HSD2) which protects the fetus from overexposure to these steroids [6]. Nevertheless, for fetuses at risk of preterm delivery, prenatal synthetic GC therapy is indispensable in stimulating the accelerated maturation of the fetal respiratory system and for the prevention of intraventricular hemorrhage, respiratory distress syndrome, and necrotizing enterocolitis, associated with preterm birth [7–9]. Current clinical recommendations point out that candidates for prenatal sGC (mainly with the use of dexamethasone) therapy are mothers at risk of premature delivery before 34 weeks of gestation. Unfortunately, glucocorticoid administration can also result in some adverse consequences on the health of both—mothers (e.g., Cushing disease, excessive body weight, hypertension, and hyperglycemia) [10–12] as well as offspring (impairment of the growth, metabolism, and physiological processes, significantly increased the risk of several diseases in adult life, e.g., osteoarthritis, hypertension, fatty liver, and diabetes) [13–17]. Moreover, the application of DEX during pregnancy could impair brain structure, and the neurogenesis process which was shown to be accompanied by adverse effects on cognition in offspring and can cause neuropsychiatric disorders, such as anxiety and depression [17, 18]. Importantly, some glucocorticoid-induced changes can be inherited by the next generations [19, 20]. Despite the importance of this issue, the mechanisms responsible for the majority of alterations appearing in the offspring’s brains after corticosteroid therapy remain unclear and are currently under intensive investigation.

One of the causes proposed to underlie adult affective behaviors such as depression and anxiety are epigenetic modifications, which are considered potential mediators of changes that are able to persist for a long time [21]. It is known that methylation marks can be stably transmitted across cell divisions and even from one generation to the next, which strongly supports the hypothesis that epigenetic modification may be a carrier of long-lasting and transgenerational effects. Susceptibility to depressive disorder may also be partly mediated by genetic factors, and hundreds of genomic variants, each with a very small effect, in this context have already been described. Genetic predisposition, in combination with/or exposure to stressful life events, can result in depression development via epigenetic modifications and therefore changes in gene expression [22]. It has been shown that chronic stress can have profound and long-lasting effects on brain gene expression responsible for the depressive phenotype [23]. Furthermore, it was suggested that long-term stress alters the epigenetic regulatory mechanisms in brain regions associated with depression such as the frontal cortex, hippocampus, hypothalamus, and the nucleus accumbens [24].

Taking all this into account, our study aimed to systematically investigate the molecular alterations that may underlie depressive-like behavior in rats, in the animal model of depression induced by prenatal dexamethasone (DEX, synthetic glucocorticoid) administration. We performed integrative, multi-level methylomic, transcriptomic, and proteomic studies. It involved reduced representation bisulfite sequencing (RRBS), to estimate the range of the genome-wide DNA methylation modifications that emerged and persisted into adulthood after prenatal DEX treatment in the frontal cortex (FCx) and the hippocampus (Hp) of these animals; the RNA sequencing, to see how this prenatal intervention may impact gene expression later in life; and finally, the data‐independent acquisition‐based quantitative proteomic analysis, to identify potential proteostasis network imbalance.

## Materials and Methods

### Animals and Treatment

The study was approved by the Local Ethics Committee in Kraków, Poland (permissions no. 211/2021 of 01.07.2021 and no. 133/2022 of 05.05.2022). In all experiments, animal suffering was minimized, and the “3R policy” was applied.

Sprague–Dawley rats were purchased from Charles River Laboratories (Sulzfeld, Germany) and kept under standard conditions which were a 12-h day/night cycle and room temperature 22 ± 2 °C, with food and water ad libitum. As described previously [25, 26], female rats with the recognized proestrus phase were placed in cages with breeding males overnight, and the presence of sperm was inspected the next morning. After confirmation of pregnancy, dams were transferred into separate cages and assigned to the experimental and control groups in a random way. The experimental animals were subjected to subcutaneous administration of the synthetic glucocorticoid, dexamethasone 21-phosphate disodium salt (DEX, Sigma‒Aldrich, Saint Louis, MO, USA, Cat. no. D1159) at a concentration of 0.13 mg/kg/ml (equal to 0.1 mg/kg DEX) dissolved in 0.9% saline, every morning from the 14th day of pregnancy until delivery. At the same time, control female rats received vehicle (saline 0.9%).

Male offspring were housed with mothers until weaning (3 weeks), then mixed within the distinct (DEX or control) groups, and housed in these groups of four to five animals per cage. The animals were sacrificed under non-stressed conditions at the age of 10 weeks, by rapid decapitation (between 9 a.m. and 12 p.m.). After decapitation, FCx and Hp were immediately dissected on ice-cold glass plates. The dissection of the FCx was carried out based on a study by Chiu et al. [27]. The whole Hp was taken for analysis. These two brain areas were chosen for the study because many lines of evidence and our previous results [25, 26] suggest that the frontal cortex and hippocampus play a substantial role in the pathophysiology of depressive disorder. Each structure was collected into separate tubes, frozen, and stored at − 80 °C, until use.

### Reduced-Representation Bisulfite Sequencing (RRBS) for Genome-Wide DNA Methylation Pattern Study

Genome-wide DNA methylation pattern study (DNA extraction, library preparation, and sequencing in the FCx and Hp, *n* = 10 per each group: DEX-treated and control rats) was outsourced to Novogene (Beijing, China) and conducted according to Meissner et al. ([28] with some modifications). Briefly, genomic DNA spiked with lambda DNA was digested with MspI, followed by end-repair and dA-tailing. Then, adapter ligation was conducted, using adapters with all cytosines being methylated. Next, samples were subjected to size selection and bisulfite conversion (0.8 µg of input DNA per sample; EZ DNA Methylation Gold Kit), which provides a modification of unmethylated cytosines into uracil, while methylated cytosines remain unchanged. Libraries were prepared using the Novogene NGS DNA Library Prep Set. The Qubit and real-time PCR measurements were applied for library quantification and the Bioanalyzer for library size distribution control. Quality-controlled and quantified libraries were pooled and sequenced on Illumina NovaSeq6000 (PE150; 9 G of raw data per sample). The data from the RRBS study have been deposited to the SRA NCBI repository, under the BioProject PRJNA1127825.

### RNA Sequencing for Transcriptome Study

RNA sequencing was carried out by Novogene (Beijing, China). Briefly, total RNA (*n* = 6–7 per group) was extracted from brain tissues and subsequently used for the reverse transcription reaction. Then, 200 ng of transcribed cDNA per sample was used for RNA library preparation with the Novogene NGS RNA Library Prep Set. The Qubit, real-time PCR, and the Agilent Bioanalyzer were applied for library quantification and library size distribution control. After cluster generation, the libraries were pooled and sequenced on a NovaSeq 6000 Illumina platform using NovaSeq 6000 S2 Reagent Kit, and 150 bp paired-end reads were generated (minimum 20 M PE reads, 6 G of raw data per sample). The data from the RNA sequencing have been deposited to the SRA NCBI repository, under the BioProject PRJNA1127825.

### Data-Independent Acquisition-Based Quantitative Proteome Study

Both selected brain areas (*n* = 7 per group: DEX-treated and control rats) were homogenized in an ice-cold lysis buffer (2% SDS, 50 mM DTT in 0.1 M Tris–HCl pH 7.6 with protease inhibitors, in a tissue to buffer ratio of 1:10 ratio (w/v)) using a TissueLyser II (Qiagen Inc., Valencia, CA, USA). Then, homogenates were boiled for 5 min at 95 °C and centrifuged (14,000 × g, 10 min) to separate undissolved residues. Finally, protein concentration was measured in each sample using the WF assay [29]. Next, a volume containing 70 µg of total protein was transferred to Microcon-30 kDa centrifugal filter units (Merck, Darmstadt, Germany), denatured with 8 M urea in 0.1 M Tris–HCl pH 8.5, and digested to peptides with use of filter-aided sample preparation (FASP) protocol [30]. Proteins were alkylated with iodoacetamide and cleaved with LysC-trypsin (Thermo Scientific, Waltham, MA, USA) with the enzyme-to-protein ratio 1:50. Digestions were carried out overnight in 50 mM Tris–HCl pH 8.5 at 37 °C. After digestion, the peptide yields were determined by WF assay, and the aliquots containing equal amounts of total peptides were desalted on C18 Ultra-Micro SpinColumns (Harvard Apparatus, Holliston, MA, USA). Samples were then concentrated to a volume of ~ 5 µl. For project-specific spectral library preparation (separately for both brain structures), an equal amount of peptides from all samples included in the analysis were combined and subjected to fractionation protocol. HpH-fractionation on C18 Micro SpinColumns (Harvard Apparatus, Holliston, MA, USA) was performed in 50 mM ammonium formate buffer (pH 10) with 12 consecutive injections of the eluent buffer, comprising 5, 10, 12.5, 15, 17.5, 20, 22.5, 25, 27.5, 30, 35, and 50% acetonitrile in 50 mM ammonium formate buffer (pH 10), collected by centrifugation (300 × g, 2 min), and dried in a centrifuge concentrator (Eppendorf, Hamburg, Germany). In this way, peptides were distributed across 12 HpH fractions separately for both subcellular fractions and analyzed by LC–MS/MS in DDA acquisition mode for library generation. Prior to the analysis, all samples and library peptide fractions were solubilized in 0.1% formic acid in 5% acetonitrile in a concentration of 0.5 µg/µl and spiked with the iRT peptide mix (Biognosys, Schlieren, Switzerland) for normalization of the retention time.

Peptides (1 µg) were injected onto a nanoEase M/Z Peptide BEH C18 75 µm i.d. × 25 cm column (Waters, Milford, MA, USA) via a trap column nanoEase M/Z Symmetry C18 180 µm i.d. × 2 cm column (Waters, Milford, MA, USA). Spectral library fractions and study samples were analyzed in DDA and DIA acquisition mode, respectively. The peptides were separated using a non-linear gradient (phase A—0.1% FA; phase B—100% ACN and 0.1% FA): 0–3.5 min, 1% B; 3.5–13 min, 1–6% B; 13–60 min, 6–15% B; 60–102 min, 15–28% B; 102–115 min, 28–36% B; 115–116 min, 36–80% B; 116–126 min, 80% B; 126–126.2 min, 80–1% B; 126.2–145 min, 1% B at a flow rate of 250 nl/min by UltiMate 3000 HPLC system (Thermo Scientific, Waltham, MA, USA) and applied to an Orbitrap Exploris 480 mass spectrometer (Thermo Scientific, Waltham, MA, USA). The nano-electrospray ion source Nanospray Flex (Thermo Scientific, Waltham, MA, USA) was equipped with a Simple Link Uno connector and LOTUS Sharp Singularity emitters (Fossil Ion Technology SL, Madrid, Spain). The ion spray voltage was set at 2.2 kV while the heated capillary temperature was 275 °C. For DDA acquisition, the mass range was set at 350–1200 m/z, the resolutions of MS1 and MS2 scans were set at 60,000 and 15,000, respectively, with a fixed cycle time (1.3 s), IT set to auto, and AGC set to 500%. For DIA acquisition, a survey scan of 120,000 resolution, IT set to auto and an AGC of 300%, was followed by 55 DIA variable m/z segments of 15,000 resolution with IT set to auto and AGC set to 1000%. The mass range was set at 350–1650 m/z, the default charge state was set to 2 and the normalized collision energy was set to 30.

The LC–MS data, library, and Spectronaut project have been deposited to the ProteomeXchange Consortium via the PRIDE partner repository [31] with the dataset identifier PXD051185. 

### Statistical Analysis

#### Methylation Study—CpG Islands Analysis

CpG island locations were downloaded from the UCSC Genome Browser using Table Browser. CpG islands were annotated with overlapping protein-coding genes (extended by 2000 bases from each side) downloaded from the Ensembl database (version 104). In a situation of multiple genes overlapping CpG island, the gene whose center was closest to CpG island was selected. CpG islands were filtered for being annotated with genes and having at least 10 cytosines with a computed methylation ratio (*n* = 13,378). For statistical analysis, two-way ANOVA with an additive model was used with treatment and cytosine site as factors.

#### Transcriptomic Data Analysis

Raw sequencing data (FASTQ files) were processed using nf-core/rnaseq v3.11.2 (10.5281/zenodo.1400710) of the nf-core collection of workflows [32] with default parameters. Data were assessed for quality and aligned to the rat reference genome, mRatBN7.2. Three samples (one ctrl and one DEX-treated in the FCx, and one ctrl in the Hp) that were greater than one standard deviation from the mean for each tissue on the first principal component (37% variance explained) of the DESesq2 variance-stabilized matrix of counts were removed from further analysis.

Differential expression analysis of gene counts was performed with the R package EdgeR v3.42.4 [33]. *p*-values were adjusted for multiple testing using the Benjamini–Hochberg method to control the false discovery rate (FDR < 0.05).

#### Proteomic Data Analysis

Project-specific spectral library generation and the experimental data analysis were conducted by Spectronaut 18 software (Biognosys) [34]. The data were filtered by 1% FDR on peptide and protein levels. Interference correction was done on the MS2 level. Protein grouping was performed based on the ID picker algorithm [35]. Peptide quantities were calculated in two modes either using the summed intensities of their respective fragment ions for MS2 or the summed isotope intensities for MS1 level. The protein CVs were calculated based on the summed intensities of their respective peptides, allowing for the differential abundance cut-off estimation, and ensuring the proper power of the statistical analysis. Data was normalized by local regression normalization, while statistical testing for differential protein abundance was done using the Spectronaut pipeline (*t*-test with multiple testing corrections after Storey [36].

#### Functional Pathway Analysis

Pathway annotations employing the KEGG database were conducted using the *Rattus norvegicus* gene base, as well as ClueGO v.2.5.6 [37, 38] and GeneMania v.3.5.2. plugins in the Cytoscape v.3.8.2 software environment [39] with accompanying the PINE tool [40] enabling organization and visualization of identified interactions. The obtained results were validated by a two-sided enrichment/depletion hypergeometric statistical test with Bonferroni step-down as a correction method for multiple testing. The kappa score threshold was set at 0.4. The analyses were conducted at the global level (cluster criteria: minimum 5 genes, constituting at least 2% of the term). The results comprised only the pathways with calculated corrected *p*-value < 0.05.

## Results

### Epigenetic Alterations Triggered by the Prenatal DEX Administration in the Adult Male Rats’ Brains

**Fig. 1:**
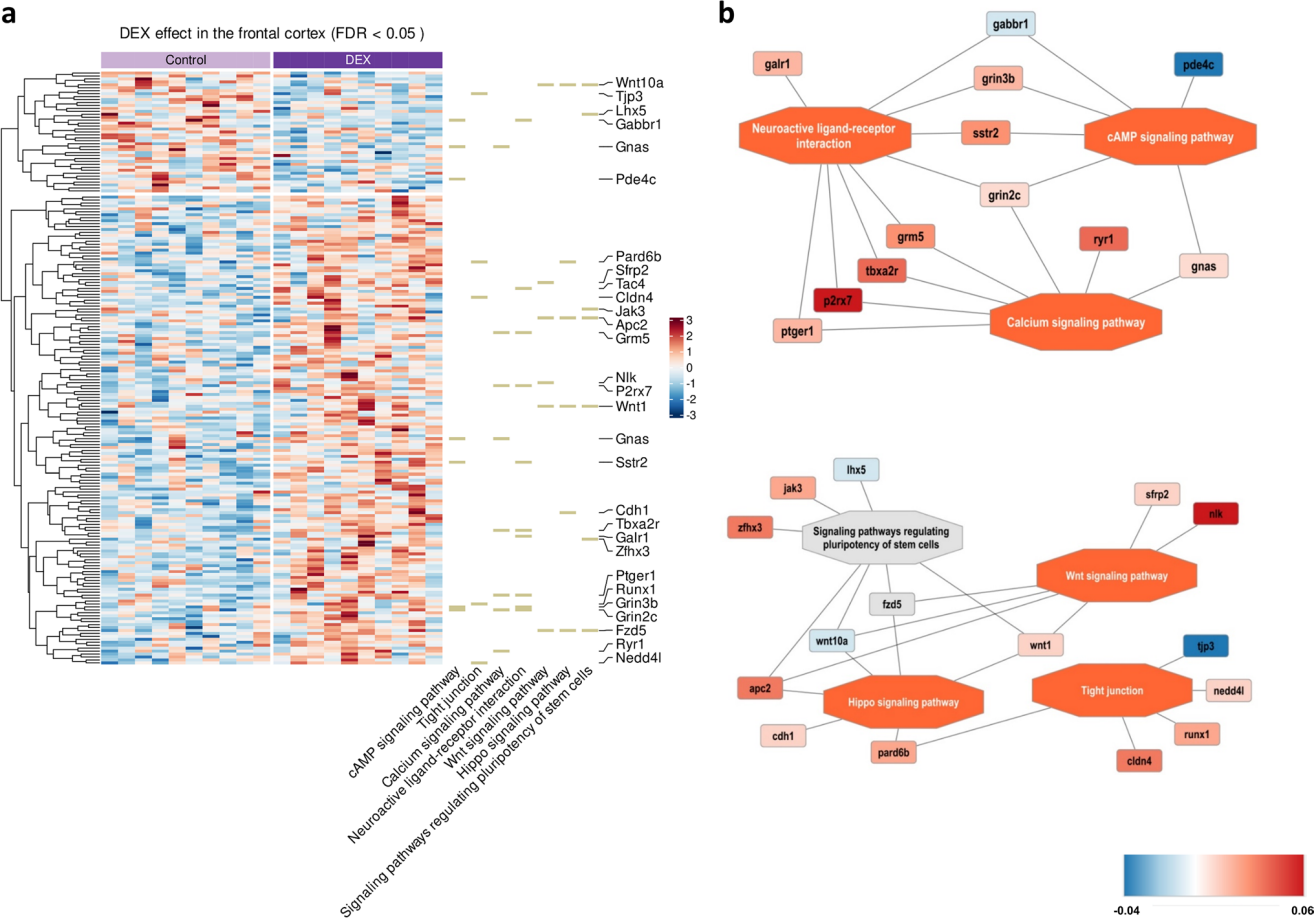
Heatmap showing the difference between groups (DEX vs control) in average CpG methylation rate in the FCx (FDR < 0.05); the intensity of the color indicates standardized methylation rate, represented as row z-scores, with specific levels detailed in the legend; KEGG terms indicated on the right with the associated genes; *n* = 10 (**a**); visualization of the enriched pathways with the associated genes and the corresponding expression level marked in color—increased methylation level of CpG—genes marked in red while decreased in blue; the legend provides the details regarding the difference between groups in average CpG methylation rate (**b**)

**Fig. 2:**
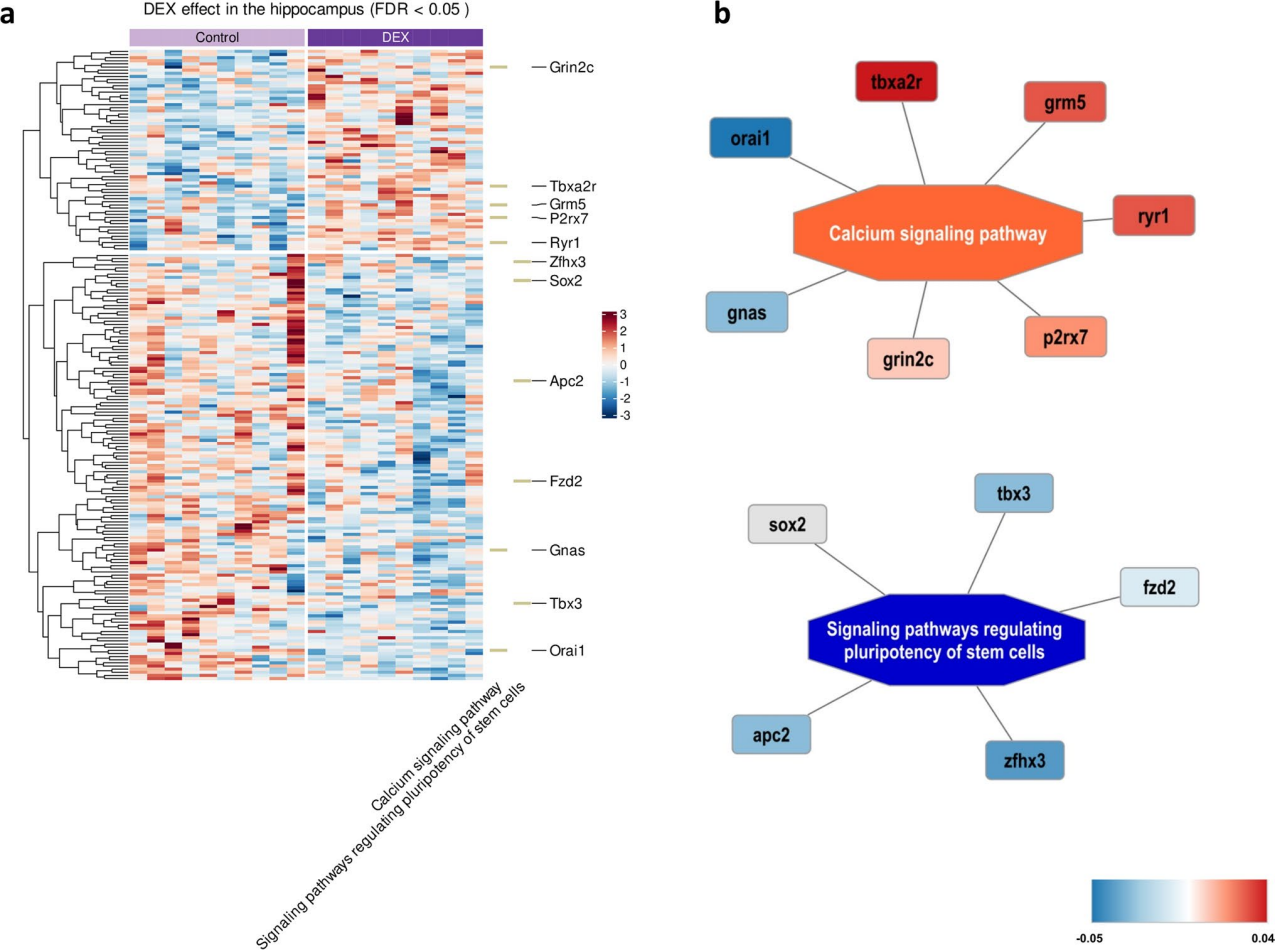
Heatmap showing the difference between groups (DEX vs control) in average CpG methylation rate in the Hp (FDR < 0.05); the intensity of the color indicates standardized methylation rate, represented as row z-scores, with specific levels detailed in the legend; KEGG terms indicated on the right with the associated genes; *n* = 10; (**a**); visualization of the enriched pathways with the associated genes and the corresponding expression level marked in color—increased methylation level of CpG—genes marked in red while decreased in blue; the legend provides the details regarding the difference between groups in average CpG methylation rate (**b**)

Modifications in the DNA methylation induced by environmental factors can alter the activity of particular genes or groups of genes maintaining the same DNA sequence and may mediate long-term effects. Therefore, we evaluated the scale of DNA methylation alterations caused by prenatal DEX administration in adult male rat’s brains. As a result, out of the 13,378 captured CpG islands that met the set conditions (being annotated with genes and having at least 10 cytosines with computed methylation ratio), we found that 200 had significantly changed methylation level in the FCx, as well as 200 in the Hp when comparing experimental animals to controls (FDR < 0.05; details in Supplementary Tables S1 and S2, respectively). Those differentially methylated CpG islands (DMIs) were annotated to 195 and 187 genes, respectively. In the FCx, 158 DMIs (79.0%) revealed higher methylation levels in the DEX-treated rats compared to the control, while only 42 (21.0%) had lower methylation levels. Interestingly, the methylation pattern of CpG islands in the Hp was far different compared to the FCx, as only 67 (33.5%) of DMIs had increased methylation levels, while 133 (66.5%) had decreased (Figs. 1a and 2a). In the FCx, the highest change we identified in CpG islands located within the exon of Dnah17 gene, encoding heavy chain associated with axonemal dynein (11.0%, *p* = 4.93E − 12), while the highest decrease in DNA methylation level was noted for DMI located in the exonic region of the Ubd gene—a ubiquitin-like protein modifier (− 8.1%, *p* = 6.53E − 21). Interestingly, the same DMIs were also the most affected in the Hp; the highest increase in the methylation level we observed for DMI localized in the Dnah17 gene (7.2%, *p* = 3.50E − 07), while the most hypomethylated CpG island was in Ubd (− 8.9%, *p* = 3.98E − 23). Top up- and down-methylated DMIs, their genomic localizations, and statistics are presented in Table 1.

**Table 1:** Top ten up and down differentially methylated CpG islands (DMIs) in the FCx and the Hp revealed in the comparative analysis of prenatally DEX-treated and control rats, the corresponding gene names, and statistics

Chr	Start	Stop	Gene symbol	Difference	SD	*p*-value	FDR
**Frontal cortex**							
10	107,057,375	107,057,797	*Dnah17*	11.0%	5.8%	4.93E − 12	1.89E − 09
4	163,119,259	163,119,551	*Clec12a*	10.6%	4.0%	6.08E − 11	1.84E − 08
12	39,395,734	39,395,948	*P2rx7*	5.9%	3.0%	2.04E − 22	1.72E − 18
1	80,495,973	80,496,585	*Clasrp*	5.7%	4.7%	1.73E − 06	2.00E − 04
19	59,873,081	59,873,614	*Tomm20*	5.3%	6.8%	1.36E − 15	1.28E − 12
1	222,673,566	222,673,815	*RGD1560108*,* Zfta*	5.0%	3.4%	8.65E − 07	1.04E − 04
10	65,963,544	65,964,104	*Nlk*	4.9%	2.5%	4.35E − 07	5.84E − 05
9	81,586,197	81,586,606	*Tmbim1*	4.8%	3.6%	2.22E − 09	5.22E − 07
8	22,056,793	22,057,052	*Icam5*	4.8%	4.1%	1.45E − 08	2.50E − 06
20	4,445,052	4,445,679	*LOC103689965, C4a*	4.7%	2.6%	1.60E − 12	6.74E − 10
20	1,897,347	1,897,638	*Ubd*	− 8.1%	4.1%	6.53E − 21	2.76E − 17
14	107,748,189	107,749,950	*Zrsr1*	− 6.2%	10.7%	3.48E − 16	3.68E − 13
7	11,310,449	11,310,696	*Tjp3*	− 4.4%	2.5%	4.98E − 04	0.026
9	97,126,327	97,126,595	*Asb18*	− 4.1%	2.1%	1.45E − 04	0.009
16	20,449,174	20,449,717	*Pde4c*	− 3.7%	2.9%	5.36E − 06	5.52E − 04
16	69,095,833	69,096,756	*Brf2*	− 3.5%	3.1%	1.71E − 10	4.65E − 08
7	12,400,455	12,401,235	*RGD1562114, Fam174c*	− 3.3%	4.4%	1.32E − 04	0.009
1	48,204,837	48,205,956	*Igf2r*	− 3.3%	1.4%	1.96E − 11	6.89E − 09
3	172,375,270	172,376,022	*Gnas*	− 3.3%	3.8%	1.34E − 04	0.009
1	71,382,958	71,383,530	*Zfp444*	− 3.2%	2.4%	2.81E − 04	0.016
**Hippocampus**							
10	107,057,375	107,057,797	*Dnah17*	7.2%	3.8%	3.50E − 07	5.02E − 05
8	46,610,854	46,611,117	*Tecta*	7.2%	3.8%	3.17E − 10	9.92E − 08
X	62,372,449	62,372,737	*Arx*	6.5%	2.4%	1.38E − 18	1.46E − 15
2	188,638,928	188,639,171	*Efna3*	5.0%	3.5%	2.04E − 06	2.40E − 04
12	16,751,236	16,751,592	*Elfn1*	4.4%	4.8%	1.43E − 04	0.009
1	221,114,368	221,115,013	*Ltbp3*	4.3%	4.1%	8.60E − 04	0.039
3	159,754,077	159,754,797	*Jph2*	4.2%	4.4%	7.73E − 06	7.78E − 04
17	30,864,293	30,864,981	*Fam50b*	4.1%	3.6%	5.29E − 05	0.004
19	59,873,081	59,873,614	*Tomm20*	4.0%	5.5%	6.76E − 11	2.38E − 08
6	137,965,379	137,965,842	*Crip1*	3.8%	2.0%	1.08E − 08	2.23E − 06
20	1,897,347	1,897,638	*Ubd*	− 8.9%	3.4%	3.98E − 23	1.68E − 19
7	14,043,175	14,043,420	*Syde1*	− 6.6%	2.9%	1.32E − 08	2.53E − 06
3	168,102,170	168,102,391	*Cyp24a1*	− 6.5%	4.2%	1.25E − 08	2.46E − 06
1	246,973,113	246,973,343	*Slc1a1*	− 6.3%	2.6%	1.16E − 07	1.86E − 05
7	11,309,856	11,310,061	*Tjp3*	− 6.0%	3.9%	7.56E − 08	1.25E − 05
9	10,632,182	10,632,444	*Ptprs*	− 5.7%	3.0%	2.20E − 14	1.33E − 11
5	153,773,746	153,774,028	*Rcan3*	− 5.5%	2.8%	9.95E − 09	2.13E − 06
11	86,519,149	86,519,403	*Septin5*	− 5.3%	4.4%	2.16E − 06	0.002
7	11,310,449	11,310,696	*Tjp3*	− 5.1%	3.0%	1.71E − 06	2.07E − 04
10	109,470,493	109,470,755	*Bahcc1*	− 5.1%	1.8%	2.69E − 21	5.69E − 18

The sets of differentially methylated CpG islands were further subjected to enrichment analysis, using the names of genes to which they were annotated. The disclosed set of differentially methylated regions was significantly enriched in genes associated with several pathways. Especially interested in the context of brain function and depressive disorder onset were the *cAMP signaling pathway*, *tight junction*, *calcium signaling pathway*, *neuroactive ligand-receptor interaction*, and *Wnt* and *Hippo signaling pathway*, as well as *signaling pathways regulating pluripotency of stem cells*. Details of the enrichment analysis are collected in Supplementary Table S3 and selected enrichments are presented in Fig. 1a, b.

In the Hp, the group of the identified hypermethylated CpG islands was significantly enriched in genes engaged in two terms: *Calcium signaling pathway* and *signaling pathways regulating pluripotency of stem cells*. Details of the enrichment analysis are collected in Supplementary Table S4 and selected enrichments are presented in Fig. 2a, b.

### Transcriptomic Alterations Induced by the Prenatal DEX Administration in the Adult Male Rats’ Brains

**Fig. 3:**
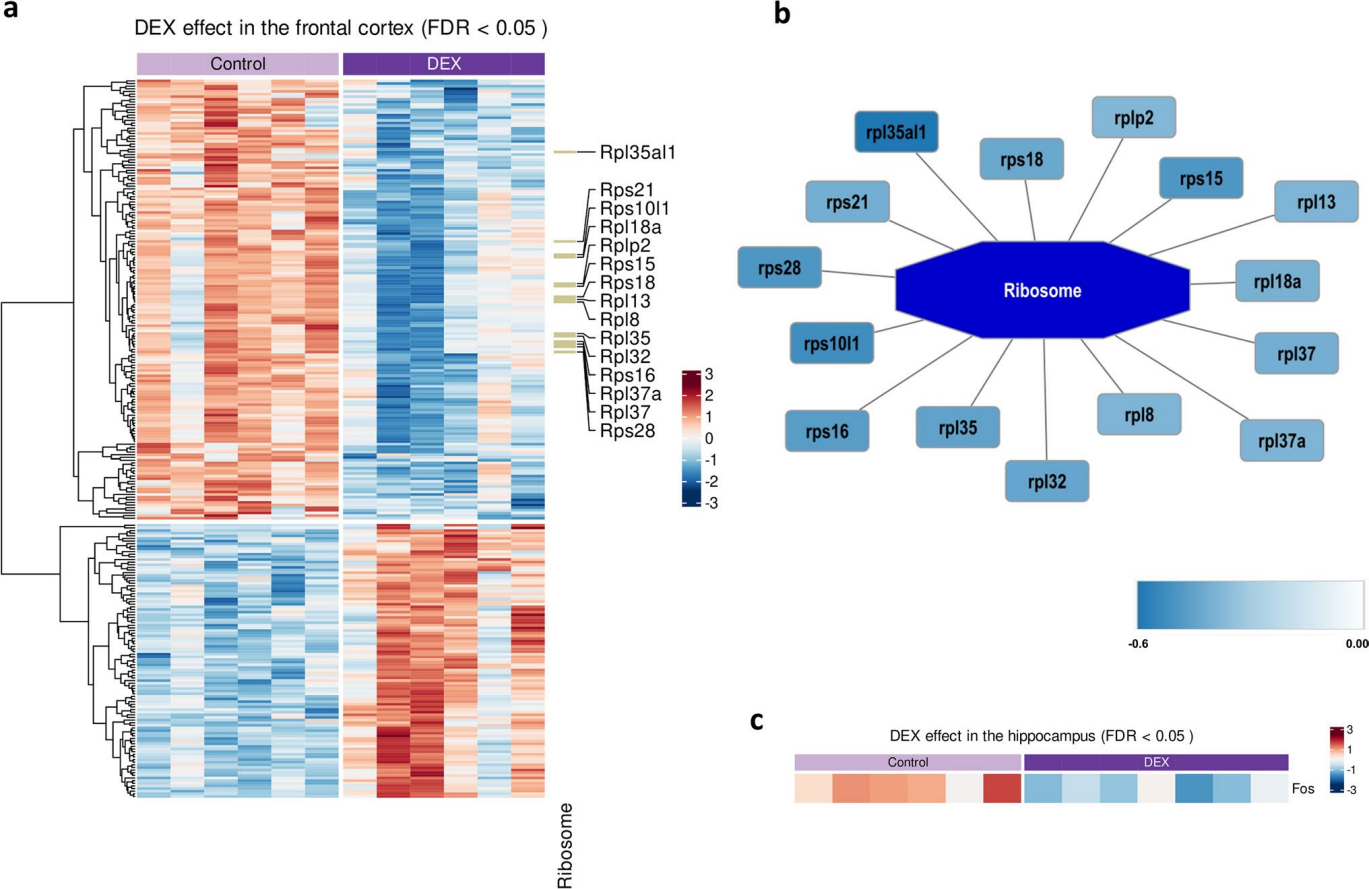
Heatmap showing differential expression of genes between DEX-treated and control rats in the FCx (FDR < 0.05); the intensity of the color indicates standardized transcript abundance levels, represented as row z-scores, with specific levels detailed in the legend; KEGG term indicated on the right with the associated genes; *n* = 6 (**a**); visualization of the enriched pathway with the associated genes and the corresponding expression level marked by color intensity—the higher the intensity, the lower the gene expression after DEX treatment; the legend provides the details regarding the magnitude (log fold change) of the observed changes (**b**); heatmap showing differential expression of genes between DEX-treated and control rats in the Hp (FDR < 0.05); the intensity of the color indicates standardized transcript abundance levels, represented as row z-scores, with specific levels detailed in the legend; *n* = 6–7 (**c**)

In the next step, we were interested in the spectrum of changes triggered by prenatal DEX administration on the transcripts level in the investigated brain regions of adult rats; therefore, RNA sequencing was performed. The data analysis revealed 271 differentially expressed genes (DEGs) in the FCx and 1 in the Hp (FDR < 0.05; details in Supplementary Tables S5 and S6, respectively). In the FCx, 104 (38.4%) of the transcripts were upregulated, while 167 (61.6%) were downregulated (Fig. 3a). The highest differences were revealed for Fam111a, Ca3, and Tmco5a (upregulated), while in the opposite were RT1-Bb, Rab1b, and Bcl3 (downregulated). Details of the top up- and downregulated transcripts are presented in Table 2. In the Hp, only one transcript, Fos, revealed differential expression, showing a down-regulation pattern in DEX-treated rats (logFC =  − 0.64; FDR = 0.032; Fig. 3c).

**Table 2:** Top ten up and down differentially expressed gene transcripts revealed in the comparative analysis of prenatally DEX-treated and control rats (FCx) and the corresponding statistics. In the Hp, only the Fos gene was found to be differentially expressed between the tested groups

	**Gene name**	**logFC**	**LR**	***p*-value**	**FDR**
**Frontal cortex**	*Fam111a*	4.02	23.55	1.22E − 06	0.004
	*Ca3*	3.29	13.07	2.99E − 04	0.034
	*Tmco5a*	1.16	11.72	6.17E − 04	0.044
	*Lrrc3c*	1.14	13.5	2.38E − 04	0.030
	*Hcrtr2*	1.01	15.19	9.74E − 05	0.022
	*Trpc4*	0.82	14.39	1.49E − 04	0.027
	*ENSRNOG00000062849*	0.77	12.07	5.12E − 04	0.043
	*Traf5*	0.76	11.47	7.07E − 04	0.046
	*Zdbf2*	0.74	11.15	8.42E − 04	0.049
	*Sstr2*	0.73	13.63	2.23E − 04	0.030
	*RT1-Bb*	− 1.61	14.64	1.30E − 04	0.026
	*Rab1b*	− 1.54	18.16	2.03E − 05	0.012
	*Bcl3*	− 1.52	13.15	2.88E − 04	0.034
	*Slc5a5*	− 1.38	26.76	2.31E − 07	0.003
	*Cd74*	− 1.37	14.14	1.70E − 04	0.028
	*RT1-Da*	− 1.36	13.21	2.78E − 04	0.034
	*Plac8*	− 1.31	19.15	1.21E − 05	0.011
	*Ddah2*	− 1.19	16.34	5.29E − 05	0.017
	*Mlkl*	− 1.19	11.58	6.68E − 04	0.044
	*P3h2*	− 1.16	11.72	6.18E − 04	0.044
**Hippocampus**	*Fos*	− 0.64	22.48	2.12E − 06	0.032

Downstream enrichment analysis revealed that among downregulated genes, 15 (namely, Rpl: Rpl13, Rpl18a, Rpl32, Rpl35, Rpl35al1, Rpl37, Rpl37a, Rpl8, Rplp2; Rps: Rps10l1, Rps15, Rps16, Rps18, Rps21, Rps28) are involved in large and small ribosomal subunits assembly, indicating that the *ribosome pathway* is disturbed in adult animals after prenatal DEX administration (Fig. 3a, b). The complete results of the enrichment analysis are collected in Supplementary Table S7. Since only one transcript was found to be differentially expressed in the Hp, the enrichment analysis in this case was omitted.

### Proteomic Alterations Resulted from the Prenatal DEX Administration in the Adult Male Rats’ Brains

To get better insight into the molecular mechanisms underlying long-lasting effects evoked by the prenatal excess of glucocorticoids, we performed data-independent acquisition analysis, allowing for a quantitative, highly sensitive, and accurate protein level assessment, to explore the proteomic landscape of the FCx and the Hp of adult rats. Compared to the genomic and transcriptomic studies, proteome analysis provides a more robust and representative picture of a functioning cell, which is a complex and highly dynamic entity [41].

DDA mass spectrometry measurements resulted in the identification of 64,949 and 66,001 proteotypic peptides, which allowed the preparation of the spectral library comprising 5180 and 5203 protein groups (including 458 and 472 single hit proteins) for FCx and Hp specimens, respectively. The libraries were then used to analyze DIA datasets with Spectronaut. The recovery of the spectral library was 83.6% and 84.4% with data completeness among protein group profiles of 95.7% and 95.1% for the FCx and the Hp, respectively. Median CVs of protein groups were calculated in the range of 5.4 to 7.3% for all experimental groups in both brain structures, which allowed for the estimation of a significant quantitative cut-off for an absolute 1.5-fold change. On average, the acquired data allowed the identification and quantification of 4191 and 4216 protein groups in FCx and Hp specimens, respectively.

As a result, we revealed a specific repertoire of differentially expressed proteins (DEPs) in two selected brain areas, including 146 DEPs in the FCx and 123 in the Hp (*q*-value < 0.01, fold change > 1.5; details in Supplementary Tables S8 and S9 including DEPs reaching post hoc statistical power of 1.0 for median CV). The majority of them had decreased levels in the experimental group compared to controls; in the FCx, 127 (87.0%) of differentially expressed proteins were downregulated, while 104 (84.6%) in the Hp. Among those with the most pronouncedly changed level of expression was upregulated Ca3, encoding carbonic anhydrase 3 (fold 16.3, *p* = 1.35E − 05), and downregulated Dnmbp, encoding dynamin-binding protein (− 5.4, *p* = 0.014). Details of the top up- and down-expressed proteins are presented in Table 3.

**Table 3:** Top ten up and down differentially expressed proteins in the FCx and the Hp, revealed in the comparative analysis of prenatally DEX-treated and control rats, and the corresponding statistics

Protein group	Protein description	Gene name	Fold change	Ratio	*p*-value	*q*-value
**Frontal cortex**						
P14141	Carbonic anhydrase 3	Ca3	16.31	16.31	1.35E − 05	1.08E − 04
Q63734	Potassium voltage-gated channel subfamily C member 4	Kcnc4	2.35	2.35	0.033	0.028
P06866	Haptoglobin	Hp	2.29	2.29	0.009	0.010
P80299	Bifunctional epoxide hydrolase 2	Ephx2	2.10	2.10	1.83E − 04	0.001
Q9QXS7	Probable N-acetyltransferase CML5	Cml5	1.99	1.99	0.016	0.016
P81718	Tyrosine-protein phosphatase non-receptor type 6	Ptpn6	1.98	1.98	0.010	0.011
P62494	Ras-related protein Rab-11A	Rab11a	1.87	1.87	0.001	0.002
Q5U1X0	Transcription initiation factor TFIID subunit 11	Taf11	1.81	1.81	0.002	0.003
Q4V8B3	Mediator of RNA polymerase II transcription subunit 24	Med24	1.74	1.74	0.044	0.035
P50116	Protein S100-A9	S100a9	1.64	1.64	0.054	0.041
M0R4F8	Dynamin-binding protein	Dnmbp	− 5.45	0.18	0.014	0.015
P04157	Receptor-type tyrosine-protein phosphatase C	Ptprc	− 4.75	0.21	1.70E − 07	6.16E − 06
Q5EAN7	Telomeric repeat-binding factor 2-interacting protein 1	Terf2ip	− 4.69	0.21	1.02E − 04	4.17E − 04
B2RYE5	Band 4.1-like protein 4B	Epb41l4b	− 4.53	0.22	0.002	0.003
O08949	Transcription initiation factor IIA subunit 1	Gtf2a1	− 4.43	0.23	8.24E − 05	3.58E − 04
P18890	Rho GTPase-activating protein 39 (Fragment)	Arhgap39	− 4.30	0.23	3.77E − 04	0.001
Q6AYZ1	Tubulin alpha-1C chain	Tuba1c	− 3.86	0.26	2.27E − 09	9.05E − 07
Q80WL2	Bystin	Bysl	− 3.11	0.32	2.27E − 04	0.001
Q6AYE5	Out at first protein homolog	Oaf	− 2.96	0.34	1.27E − 05	1.04E − 04
Q6AY46	tRNA (adenine(58)-N(1))-methyltransferase catalytic subunit TRMT61A	Trmt61a	− 2.95	0.34	0.001	0.002
**Hippocampus**						
P14141	Carbonic anhydrase 3	Ca3	5.70	5.70	9.8E − 05	5.0E − 04
P06866	Haptoglobin	Hp	3.25	3.25	0.002	0.004
Q8K4Y7	Soluble calcium-activated nucleotidase 1	Cant1	2.45	2.45	9.7E − 05	4.9E − 04
O88267	Acyl-coenzyme A thioesterase 1	Acot1	2.44	2.44	1.2E − 04	5.8E − 04
Q710E6	SUN domain-containing ossification factor	Suco	2.23	2.23	0.004	0.007
Q6AYR4	Torsin-2A	Tor2a	2.21	2.21	0.049	0.043
P80299	Bifunctional epoxide hydrolase 2	Ephx2	2.03	2.03	6.0E − 04	0.002
Q0V8T6	Contactin-associated protein like 5–1	Cntnap5a	1.93	1.93	0.050	0.043
D3ZSK5	N-lysine methyltransferase SETD6	Setd6	1.86	1.86	0.014	0.016
P02683	Neuronal vesicle trafficking-associated protein 1	Nsg1	1.84	1.84	1.7E − 04	7.2E − 04
Q8R515	Zinc fingers and homeoboxes protein 1	Zhx1	− 4.33	0.23	0.058	0.049
Q8K3Y6	Zinc finger CCCH-type antiviral protein 1	Zc3hav1	− 4.10	0.24	0.003	0.005
B1H222	RAB6-interacting golgin	Gorab	− 3.82	0.26	0.001	0.003
G3V8V5	Potassium channel subfamily K member 4	Kcnk4	− 3.74	0.27	2.06E − 08	5.72E − 06
Q63921	Prostaglandin G/H synthase 1	Ptgs1	− 3.73	0.27	0.002	0.004
Q9JHE5	Sodium-coupled neutral amino acid symporter 2	Slc38a2	− 3.50	0.29	0.001	0.003
Q06000	Lipoprotein lipase	Lpl	− 3.42	0.29	0.046	0.040
Q32Q05	Ubiquitin thioesterase OTU1	Yod1	− 3.42	0.29	1.41E − 06	3.72E − 05
Q66HF2	Transmembrane 9 superfamily member 1	Tm9sf1	− 3.31	0.30	0.020	0.022
P06302	Prothymosin alpha	Ptma	− 3.28	0.30	2.20E − 05	1.91E − 04

**Fig. 4:**
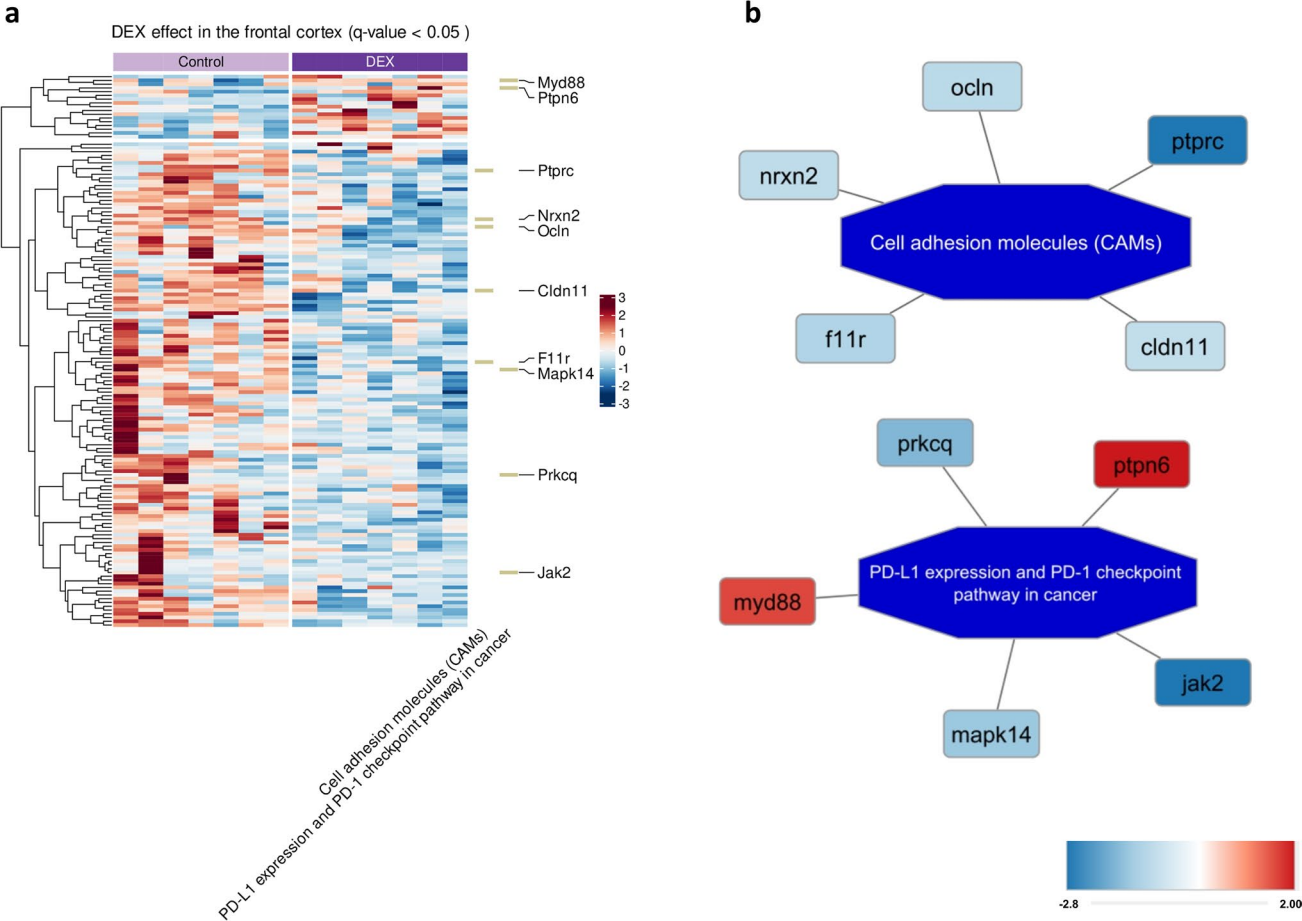
Heatmap showing differential expression of proteins between DEX-treated and control rats in the FCx (*q*-value < 0.05); the intensity of the color indicates standardized protein abundance levels, represented as row z-scores, with specific levels detailed in the legend; KEGG terms indicated on the right with the associated genes; *n* = 7 (**a**); visualization of the enriched pathways with the associated proteins and the corresponding expression level marked in color—proteins marked in red had increased expression level while decreased in blue; the legend provides the details regarding the magnitude (fold change) of the observed changes (**b**)

**Fig. 5:**
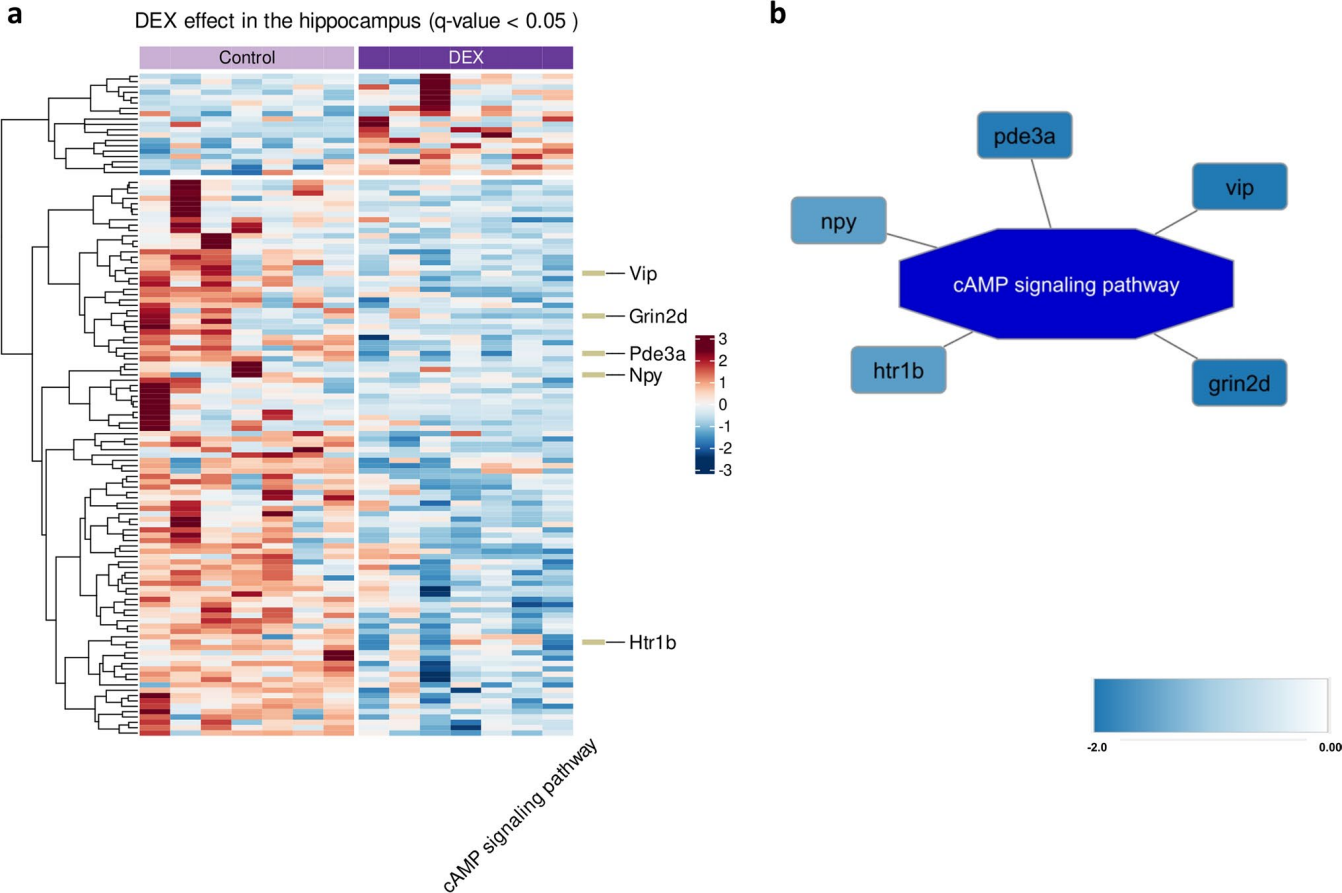
Heatmap showing differential expression of proteins between DEX-treated and control rats in the Hp (*q*-value < 0.05); the intensity of the color indicates standardized protein abundance levels, represented as row z-scores, with specific levels detailed in the legend; KEGG term revealed by enrichment analysis indicated on the right with the associated genes; *n* = 7 (**a**); visualization of the enriched pathway with the associated proteins and the corresponding expression level marked in color—the higher the intensity, the lower the gene expression after DEX treatment; the legend provides the details regarding the magnitude (fold change) of the observed changes (**b**)

The identified DEPs were used for further enrichment analysis and, among the most interesting pathways, unclosed *cell adhesion molecules* (CAMs) and *PD-L1 expression* and *PD-1 checkpoint pathway in cancer* pathways depicted in Fig. 4a, b. In the Hp, only one—the *cAMP signaling pathway*—was found to be significantly enriched (Fig. 5a, b). Details of the enrichment analysis are collected in Supplementary Tables S10 and S11.

## Discussion

In the present study, we applied a multi-level approach involving methylomic, transcriptomic, and proteomic studies of rats’ brain tissues—FCx and Hp—aiming to identify factors and characterize their engagement in molecular pathways potentially altered in individuals prenatally treated with glucocorticoids. As previously described [25, 26], our research was conducted in an animal model of depression, the principle of which involves the administration of synthetic glucocorticoid (DEX) to pregnant females from the 14th day of pregnancy until delivery (see details in the “Materials and Methods” section). Prenatal exposure to DEX induces behavioral deficits expressed as the extended immobility time shown in the forced-swim test and anxiety behavior, a symptom common in depression, demonstrated by a decrease in the open arm entries and a reduction in the time spent in open arms in the elevated plus maze test as well as anhedonia [18, 25]. Moreover, our group also demonstrated that prenatal treatment with DEX generates deficits in memory processes (impairment of spatial and recognition memory) [26]. Nevertheless, the exact mechanism underlying these pathological consequences is still unclear. Epigenetic alterations are suspected as one of the possible mechanisms responsible for the long-lasting effects of adverse factors acting early in life, and some studies have already investigated this hypothesis (for review, see [42]).

In the current study, we revealed alterations in the methylation status of numerous genes, some of which are essential for the proper development and functioning of the brain. In particular, we focused on the CpG islands’ methylation status, which provides a more informative picture of the epigenetic regulation of gene expression compared to whole genes methylation level. Nevertheless, it should be noted that predicting the inherent modulatory effects of methylation changes (especially in non-promoter regions) on gene expression remains challenging. In our study, we identified 200 differentially methylated CpG islands in the FCx when comparing DEX-treated and control animals, and 200 in the Hp. Enrichment analysis revealed several pathways associated mostly with intracellular signal transduction and ligand-receptor interactions. In the FCx, among the most important glucocorticoid-driven effects was alteration in the *cAMP signaling pathway.* cAMP, 3′,5′-cyclic adenosine monophosphate, is generated from adenosine triphosphate (ATP) by G-proteins that are associated with metabotropic receptors and can be released upon receptor activation. cAMP is a crucial signaling molecule involved in various cellular processes in the central nervous system (CNS), regulating i.a. synaptic plasticity, neuronal growth and development, and memory consolidation as well as glucose and lipid metabolism [43]. Since it is known that metabolic disturbances in the brain may be responsible for the onset of depression and astrocytes play a pivotal role in the supplying of energy substrates to neurons, astrocytic cAMP may regulate glycogenolysis and lactate release, whose level was found to be elevated in the FCx of prenatally DEX-treated rats, as we have shown previously [25]. We also demonstrated that a reduction in the oxidative phosphorylation process and in consequence diminished ATP synthesis may result from the reduced transport of lactate from astrocytes to neurons [25], and, as we observed in the current research, it can also be the consequence of alterations in the methylation status of genes involved in the cAMP signaling pathway. Moreover, among the genes characterized by the upregulated methylation pattern was Tomm20 which encodes mitochondrial outer membrane protein, mitophagy, and mitochondria abundance marker. Also in the Hp, Tomm20 was among the 10 genes with the most hypermethylated CpG island, which may further confirm the important role of dysregulation of mitochondrial function in the brain in the pathogenesis of depression induced by an unfavorable factor acting in the perinatal period. Therefore, it also seems that DEX may epigenetically regulate mitochondrial proteins’ gene expression and thus influence brain bioenergetics and energy production processes, which was documented earlier by us [25, 26]. Additionally, the dysregulation of the cAMP/Ca^2+^ signaling pathway is under debate as to whether it may be the missing link between brain insulin resistance and cognitive dysfunctions. Although scarce, there is data supporting this hypothesis, (reviewed in [44]). Our results also indicate the association between abnormalities in the regulation of the cAMP level, insulin signaling pathway, and cognitive disturbances (what we observed also under the influence of DEX) [25, 26]. It seems that also the control of neurotransmitters/hormone release could be accomplished through modifications of the cAMP pathway. In the current study, we found deregulation of methylation pattern in five genes involved in cAMP signaling, including genes encoding G protein-coupled receptor GABA (B1)—*Gabbr1* and Somatostatin—*Sstr2*; phosphodiesterase *Pde4c*; and ionotropic glutamate NMDA receptor subunits *Grin2c*, *Grin3b*. The balance between factors catalyzing the formation of cAMP (adenylate cyclase) and its degradation (phosphodiesterases) determines the level of cellular cAMP. This in turn regulates the activity of downstream pathways, which at the final steps leads to the activation of *N*-methyl-d-aspartate (NMDA) receptors, playing a critical role in, e.g., synaptic plasticity. Importantly, a significantly lower level of cAMP was found in the brains of patients suffering from depression, when compared to control, healthy individuals [45], while this effect was reversed after selective serotonin reuptake inhibitor (SSRI) treatment [46]. Glutamatergic neurotransmission dysfunction, especially by NMDA receptors and changed expression of NMDA receptor subtypes, which leads to impaired NMDA receptor-mediated intracellular signaling pathways, contributes to the pathophysiology of major depressive disorder [47]. We found that prenatal GC exposure affected the methylation of genes encoding glutamate receptors and their subunits (e.g., *Grin2c*, *Grin3b*, *Grm5*) in the FCx and Hp. It was shown that the cAMP pathway in astrocytes increases the expression of glutamate transporters, but on the other hand, acute cAMP increase may lead to downregulation of glutamate uptake by astrocytes [48]. In fact, glutamate receptors play a crucial role in many processes including synaptic development, plasticity, learning, and memory, as well as in synapse maturation and synaptogenesis throughout life [47] and any interference in their function may lead to the development of neurological or neuropsychiatric disorders. However, not only dysfunction in the glutamatergic excitatory system but also an imbalance in excitatory and inhibitory neurotransmission (in which γ-aminobutyric acid, GABA, is involved), which are reported in humans and animal models and display depressive-like symptoms [49], seems to be epigenetically regulated. GABAB receptors modulate the activity of voltage-dependent calcium channels and currents, and their activation results in the inhibition of these channels and suppression of neuronal activity. Targeting GABAB receptor activity has been identified as a valuable therapeutic approach in depression and anxiety since the antidepressant effect of GABAB receptor antagonists (i.e., ketamine, Ro-25–6981) was shown [50–53]. In our study, we found decreased methylation levels in the promoter region of the *Gabbr1* gene. These changes indicate a possible cause through which the behavioral cognitive alterations (impairment of spatial and recognition memory) that we observed in the animal model of depression may occur [25, 26]. The fact that modulation of the cAMP pathway plays a significant role in regulating cognitive processes is strongly supported by our observation that this pathway was strongly downregulated also at the protein level in the Hp—the structure most closely related to memory and learning processes. Furthermore, in both studied brain areas, the most hypermethylated CpG island was enclosed to the gene Dnah17 encoding microtubule-associated motor protein complexes. In neurons, microtubule function is crucial for axon structure and transport of neurotransmitter receptors, synaptic vesicle precursors, and mRNAs over long distances. It is also needed for an adequate response to cellular and environmental stress and according to this, dysregulation of motor activity may affect diverse neuronal functions [54].

Moreover, in the Hp, we also observed the downregulation of the transcript, an immediate-early gene (IEG) encoding c-fos. In neurons, c-fos expression appears to be stimulated by cAMP and Ca^2+^ through the activation of the CREB/CRE complex. IEGs play a crucial role in the interactions between genes and the environment because they control the expression of a wide range of genes, allowing for a long-lasting and sustained adaptation by providing the molecular basis for a rapid and dynamic response to neuronal activity. IEGs are utilized as a marker to understand neuronal ensembles associated with the formation of certain memories; they are essential to facilitate memory learning and storage [55]. The exposition to a learning paradigm or induction of long-term potentiation (LTP) results in enhanced IEG expression right following the treatment [56].

The findings from the present study showed the altered methylation pattern in genes encoding channels contributing to the calcium signaling pathway in both examined brain areas. Calcium ions (Ca^2+^) play a fundamental role in neurotransmission by facilitating the release of neurotransmitters, influencing postsynaptic responses, participating in various intracellular signaling pathways, and contributing to the overall regulation of synaptic communication as well as participating in astrocyte-neuron signaling [57, 58]. Astrocytic Ca^2+^ elevation induces glutamate (but also ATP, GABA, and d-serine) release from astrocytes in a process called “gliotransmitter release,” while these gliotransmitters mediate further neuronal excitation or inhibition [59]. Furthermore, a very recent study concerns the role of the Orai1 calcium channel, whose gene *Orai1* was also one of those differentially methylated in the Hp in our study. It was shown that deletion of this channel in astrocytes leads to decreased expression of genes involved in inflammation, metabolism, and cell cycle pathways, as well as reduction in cellular metabolites and ATP production [60]. In the Hp, loss of *Orai1* attenuates inhibitory neurotransmission and inflammation-induced astrocyte Ca^2+^ signaling, while in *Orai1* knockout mice amelioration of LPS-induced depression-like behaviors including anhedonia and helplessness were reported [60].

Enrichment analysis of differentially methylated regions in the FCx unclosed also *Wnt* and *Hippo signaling pathways* to be affected by prenatal DEX treatment. In the adult brain, increasing evidence suggests that the Wnt pathway plays an essential role in the regulation of structure and function of the nervous system, synaptic transmission, and neurogenesis, while dysregulation of this pathway has been associated with brain disorders, such as Alzheimer’s disease and mood disorders [61, 62]. Although the Wnt signaling pathway has been intensively studied, also in the context of excess glucocorticoid exposure, data concerning prenatal glucocorticoid exposition are sparse. It is known that knock-down of Wnt pathway-associated genes led to alterations in the Wnt/β-catenin signaling, neurogenesis deficits, and depression-like behavior, which was reversed upon Wnt overexpression [63]. Also, other studies point to the involvement of Wnt signaling in the regulation of new neuron development and cognitive function [64]. Furthermore, components of the Wnt pathway are transcriptional and downstream targets of the Hippo pathway; epigenetically induced overexpression of the Hippo pathway and disruption of cellular processes involved in learning and memory are associated with an increased risk of stress-related psychiatric disorders [65]. The Hippo signaling pathway has been described as a key regulator of tissue growth, which in the CNS regulates proliferation, differentiation, and regeneration of neurons, neural progenitor cells, and neural stem cells (NSCs), but also plays a role in synaptic development [65–68]. To the best of our knowledge, our study is the first that associates prenatal glucocorticoid treatment with alterations in the Hippo signaling pathway-related gene methylation in adulthood. Because it is still poorly investigated, this aspect should be addressed in further studies to establish an extended explanation for this finding.

What is important, in our study, in the FCx is that genes clustered in *Tight junction* terms, which are responsible for cell–cell adhesion complex formation between brain endothelial cells in order to establish a barrier to limit the free flow of molecules between the blood and brain, were hypermethylated. Disruption of the blood–brain barrier (BBB) tight junctions and dysregulation of the barrier integrity is a common pathology reported in major psychiatric disorders with the most severe effects observed in patients diagnosed with depression and schizophrenia [69]. Functional and structural changes in the BBB endothelium, loss and mislocalization of tight junction proteins, and reduced astrocyte coverage contribute to BBB breakdown during the depression. Impaired endothelial function and BBB disintegration lead to cerebral perfusion deficiencies, which causes brain injury as well as emotional and cognitive issues. Loss of cerebrovascular integrity and BBB degradation can lead to infiltration of immune cells, activation of glial cells, and the production of a variety of inflammatory mediators and ROS, resulting in neuroinflammation and neuron death (reviewed in [70]). Our study demonstrated that these detrimental effects can be induced already at the stage of epigenetic programming.

Enrichment analysis indicates also that in both examined brain structures, methylation patterns were changed in genes classified into group *signaling pathways regulating pluripotency of stem cells.* It is currently assumed that neurodifferentiation in the adult brain occurs in specific niches of the subventricular zone of the lateral ventricles and the subgranular zone of the dentate gyrus of the Hp and at a very low level in cortical neurons. Stem cells are generally considered to be glycolytic and in agreement with this also neural stem cells (NSCs) in the brain are considered to have predominantly glycolytic activity. During neurogenesis, NSCs proliferate to become neural progenitor cells (NPC) which turn into mature neurons. A variety of modifications occurred during this process, such as the proliferation and activation of mitochondria, and the switch from glycolytic to aerobic metabolism, which are needed to adapt to changing metabolic demands. The activation of stem cells coincides with changes in mitochondrial morphology and function, which are believed to be fundamental for the proper neurogenesis process (reviewed in [71]). Therefore, it seems that the serious metabolic alterations that we observe in the brain, in the studied model of depression, may have their basis at the level of epigenetic regulation which may contribute to the disturbances in the process of adult neurogenesis. Simultaneously, NSCs are highly influenced by monoaminergic neurotransmitters and cognitive deficits that accompany depression and may also be exacerbated by decreased neurogenesis.

Alterations in the methylation pattern of genes related to the identified signaling pathways in both FCx and/or Hp indicate a highly possible mechanism driving the long-term consequences of prenatal glucocorticoid exposure on the brain persisting into adulthood, but additional studies were required to better understand the machinery of these modifications. Therefore, to deliver additional insights into the studied glucocorticoids’ long-term effects, the next step of our study involved the evaluation of the global transcriptome level. As a result of the applied treatment, we found 271 differentially expressed transcripts in the FCx of adult rats, among which further analysis clearly showed a significant number of genes associated with large and small ribosomal subunit assembly. Ribosomes are principal components of the protein synthesis machinery but ribosomal dysregulation in the pathophysiology of depression has been identified just recently. A comparative transcriptomic study, involving brain tissue collected from postmortem subjects with diagnosed depression and from rodents exposed to chronic variable stress demonstrated, in line with our results, down-regulation of ribosomal protein genes (RPGs) in both—humans and mouse models [72]. Moreover, an in vitro study showed that deregulation of the ribosomal protein gene expression was a glucocorticoid-driven response to stress. It has been established that post-mitotic neurons possess vast amounts of ribosomes and depend on controlled translation to develop and maintain their phenotypic features, such as neurite and synaptic morphogenesis, and additionally synaptic plasticity. The downregulation of RPGs in the brain can result in reduced ribosome biosynthesis throughout the neurons, leading to a global decrease in translation and protein synthesis in these cells. Moreover, since ribosomes are involved in the synthesis of transmitter receptors, synaptic scaffolding proteins, and other regulatory factors, the observed changes may indirectly alter the neurotransmission process. In addition to the above-mentioned effects, downregulation of RPG expression may alter ribosome composition in specific cellular locations which can be observed as the removal, modification, or substitute of a few RPs. Such changes have the potential to produce specialized ribosomes, altering the translation of, e.g., synaptic proteins, in a compartment-specific way (reviewed in [73]).

In line with this are data collected from proteomic analysis. They suggest that in both brain structures, prenatal DEX administration triggers a long-term decrease in the expression of a significant number of proteins. Moreover, although most of the DEPs identified in this study were downregulated, we also found important results regarding proteins whose expression was upregulated as a result of prenatal glucocorticoid overexposure. A drastic increase, not only in the protein but also in the transcript level, was detected for the carbonic anhydrase type III (Ca3) (protein level fold change = 16.3, mRNA level logFC = 3.1). Carbonic anhydrases (CAs) are a family of zinc metalloenzymes that catalyze the reversible hydration/dehydration of CO_2_/HCO_3_ which makes them highly important during processes, which require maintaining the appropriate cellular pH level [74]. The catalytic activity of the Ca3 isoform for the CO_2_ hydration process is quite low, but recently it was shown that cytosolic CAs can be involved in acid–base balance, respiration, carbon dioxide, and ions transport, and increasing evidence suggests their involvement in the pathogenesis of various disorders [75]. Among the cytosolic CA isoforms, Ca3 is an enzyme that is mainly present in tissues characterized by a high oxygen consumption rate, such as skeletal muscle, liver, and brain but it possesses a different, yet unknown physiological function. Pharmacological studies (on topiramate and acetazolamide) indicate that CA inhibitors, which are already used in clinics, may impair memory and, on the other hand, administering CA activators to animal models improves learning and memory [76].

In addition to the above-mentioned signaling pathways that we showed to be altered at the protein level, we also demonstrated significant modifications in several others—relevant in the context of depressive-like changes—molecular pathways. Currently gaining great interest among researchers is the PD-1/PD-L1 pathway. The immune system inhibition by the interaction of the PD-1 receptor on the immune cells with PD-L1 expressed on the surface of cancer cells is known as one of the key mechanisms allowing cancer cells to elude the immune response and subsequently promote tumor growth through immune tolerance. So far, changes in checkpoint regulation have been also observed in other disorders such as brain tumors, Alzheimer’s disease, ischemic stroke, spinal cord injury, and multiple sclerosis, but to date, there is only a few data on the immune checkpoints (ICP) involvement in the course of neuropsychiatric disorders. It has been demonstrated that the development of learned helplessness in mice is caused by Th17 cells found in the Hp, which over-express PD-1 [77]. Moreover, it was found that aging in C57BL/6 J mice is linked to elevated PD-1 expression in FCx and hypothalamic microglial cells, which could potentially exacerbate emotional and motor discoordination associated with aging [78]. Furthermore, PD-L2 expression on monocytes was found to be lower in bipolar disorder patients compared to healthy controls, according to Wu et al. [79]. Also, our unpublished data shows that PD-L1 expression is significantly decreased in the rat model of treatment-resistant endogenous depression and model of depression based on prenatal stress procedure, in the same brain structures as we examined in this study. Therefore it seems to be justified to hypothesize that the interaction of the immune system and the nervous system takes place at the ICP level.

Finally, at the protein level, we found the downregulation of the *cell adhesion molecule* (CAMs) pathway in the FCx. This pathway plays a key role during the formation of tissues and organs, as well as higher-order functions of living organisms such as neuronal communication. Also, contact between synapses in the CNS during neurotransmission is a specific type of adhesion, which is facilitated by CAMs. Through protein–protein interaction signaling cascades, CAMs actively control the forming of new synapses and modify the functioning of existing synapses via cell-to-cell connections [80]. Synaptically localized adhesion molecules can affect synaptic development, dendritic spine formation, synaptic receptor function, and synaptic plasticity. There are four main families of CAMs in the brain—cadherins, integrins, selectins, and immunoglobulin superfamily of cell adhesion molecules (IgSF). Integrins regulate spine function by controlling receptor trafficking in a subunit-specific manner, while cadherins control the formation of spines and synapses in excitatory neurons. CAMs are not only responsible for changes in synapses and network connectivity but they are also associated with permanent changes in physical forces throughout time, causing changes in brain plasticity. Knocking down the gene encoding α-integrin in *Drosophila melanogaster* leads to inhibition in short-term olfactory learning, which demonstrates the impact of integrins on long-term plasticity. These factors can also regulate LTP by affecting the activity of NMDARs and AMPARs. Cadherins modulate synapse activity after NMDA activation. However, their action is required for LTP and spine expansion, but not for long-term depression (LTD), spine density, or morphology, indicating their role in synaptic plasticity. IgSFs also control synapses by crosslinking to NMDARs and CaMKII through postsynaptic scaffolds (reviewed in [81]). In our study, we also showed that the most downregulated protein in the FCx was Dnmbp (more than 5 × decrease), a scaffold protein regulating actin cytoskeleton and synaptic vesicle pools, which also can escalate potential changes in synapses. In connection with this, in this study we also found hypermethylation of CpG islands of the Icam5 gene, encoding protein expressed on the surface of neurons, displaying adhesion activity and impacting functional synapse formation. This proves that adhesion proteins (also involved in neuron-microglial cell interactions) play a role not only in the course of normal development but also in pathological conditions.

## Conclusions and Future Directions

Here we presented the first comprehensive, multi-omic (methylomic, transcriptomic, and proteomic) analysis of the effects triggered by the prenatal DEX administration in the FCx and the Hp of adult male rats. Our findings provide new, extensive insights into the changes that may underlie depressive behavior and cognitive impairment found in animals exposed to prenatal excess of GCs. It shows that the DEX administration, besides its beneficial effects, has also the potential to inconveniently modulate numerous signaling pathways in the developing fetal brain, which may have long-term consequences in adulthood. Nevertheless, further research in this field is necessary to better understand complex intra- and intercellular deregulations in CNS leading to the long-term effects of GCs, and their contribution to the onset of depressive disorder. The observed changes in memory and synaptic plasticity induced by DEX action advocate the use of electrophysiological techniques and diverse approaches, including optogenetic tools, in addition to the traditional measurements of excitatory postsynaptic currents and paired-pulse ratio, to improve the characterization of the contribution of pre- and postsynaptic mechanisms. Also, research on mitigating the observed behavioral alterations through various treatment strategies will directly indicate whether the changes we have observed in the brain at multiple molecular levels are crucial in depressive disorder pathomechanisms.

## Limitations of the Study

The main limitation of our study covers the inclusion of male animals only, which should be taken into account when interpreting the data, as sex-specific differences in stress and glucocorticoid response between males and females are known. Moreover, for the DNA methylation level examination, we applied the RRBS method, which protocol generally identifies DMRs in higher CpG density regions of ≥ 3 CpG/100 bp which corresponds to approximately 20% of the genome [82]. WGBS would be a promising alternative, as this method generally identifies ≥ 2 CpG/100 bp, which corresponds to approximately 50% of the genome [82].

## Supplementary Information

Below is the link to the electronic supplementary material.

Supplementary file1 (XLSX 151 KB)

## Data Availability

All raw fastq files (RRBS and RNA-Seq data) have been deposited to the SRA NCBI repository, under the BioProject PRJNA1127825. The LC–MS data, library, and Spectronaut project have been deposited to the ProteomeXchange Consortium via the PRIDE partner repository [31] with the dataset identifier PXD051185. Analyzed data is provided within the supplementary file.
